# Post-COVID-19 Pulmonary Fibrosis

**DOI:** 10.7759/cureus.22770

**Published:** 2022-03-02

**Authors:** Asma Mohammadi, Irina Balan, Shikha Yadav, Wanessa F Matos, Amrin Kharawala, Mrunanjali Gaddam, Noemi Sarabia, Sri Charitha Koneru, Siva K Suddapalli, Sima Marzban

**Affiliations:** 1 Public Health, University of Nebraska Medical Center, Omaha, USA; 2 Research and Academic Affairs, Larkin Community Hospital, Miami, USA; 3 Internal Medicine, State Medical and Pharmaceutical University "N.Testemitau", Fayetteville, USA; 4 Internal Medicine, Kathmandu University, Kathmandu, NPL; 5 Research, Institute of Systems Biology (ISB) - Hadlock Lab, Seattle, USA; 6 Medicine, Medical College Baroda, Vadodara, IND; 7 Internal Medicine, Jacobi Medical Center/Albert Einstein College of Medicine, New York City, USA; 8 Internal Medicine, Andhra Medical College, Visakhapatnam, IND; 9 Internal Medicine, Mayo Clinic, Rochester, USA

**Keywords:** covid-19, ards, lung fibrosis, sars cov and pulmonary fibrosis, covid-19 and pulmonary fibrosis

## Abstract

Severe acute respiratory syndrome coronavirus 2 (SARS-CoV-2) has infected millions worldwide with a high mortality rate due to a lack of definitive treatment. Despite having a wide range of clinical features, acute respiratory distress syndrome (ARDS) has emerged as the primary cause of mortality in these patients. Risk factors and comorbidities like advanced age with limited lung function, pre-existing diabetes, hypertension, cardiovascular diseases, and obesity have increased the risk for severe COVID-19 infection. Rise in inflammatory markers like transforming growth factor β (TGF-β), interleukin-6 (IL-6), and expression of matrix metalloproteinase 1 and 7 (MMP-1, MMP-7), along with collagen deposition at the site of lung injury, results in extensive lung scarring and fibrosis. Anti-fibrotic drugs, such as Pirfenidone and Nintedanib, have emerged as potential treatment options for post-COVID-19 pulmonary fibrosis. A lung transplant might be the only life-saving treatment. Despite the current advances in the management of COVID-19, there is still a considerable knowledge gap in the management of long-term sequelae in such patients, especially concerning pulmonary fibrosis. Follow up on the current clinical trials and research to test the efficacy of various anti-inflammatory drugs is needed to prevent long-term sequelae early mortality in these patients.

## Introduction and background

The novel coronavirus is an enveloped single positive-stranded virus (+ssRNA) with spikes of glycoproteins on the outer layer, distinguishing it from the Coronaviridae family [1]. Reports of people infected with the severe acute respiratory syndrome (SARS) originated in Wuhan, China, in December 2019 [2,3]. COVID-19 was declared a global pandemic on March 11, 2020 [4]. As of February 15, 2022, the coronavirus cases transcended 77,025,050 confirmed cases in the United States (US) [5].

Indeed, the development of a vaccine is a significant accomplishment in human history [6]. However, evidence shows that the long-term adverse consequences of COVID-19 patients can be a considerable health complication for the people who have recovered [7]. Multiple studies now indicate that increased risk of pulmonary fibrosis followed a severe COVID-19 infection and is mainly observed in patients with comorbidities such as diabetes, hypertension, or coronary disease [8]. In addition, many researchers described that the inflammatory process generated could lead to lasting structural changes in the lungs, such as fibrosis [9].

The classification is based on COVID-19 infection severity [10]. Stage 1 includes mild symptoms (flu-like illness), cough, cold, fever, sore throat, myalgias, body aches, and headache. In the second stage, pulmonary inflammation and coagulopathy can occur, presenting as dyspnea and hypoxemia [11,12]. Most (about 49%) of the severe cases ended in acute respiratory distress syndrome (ARDS) and venous thromboembolism [9,11]. Pulmonary fibrosis can be one of the complications of severe infection seen in the third stage [11].

It has been reported that asymptomatic patients carry similar infectivity as symptomatic infections [13]. COVID-19 infection could lead to an inflammatory response, including the cytokine storm and other various regulatory pathways to counteract the damaged tissue [14]. The virus has the potential of binding to the angiotensin-converting enzyme 2 (ACE2) receptors on the upper respiratory tract [14], which will increase the concentration of angiotensin 2, causing the activation of interleukin-6, tumor necrosis factor-α, recruitment of neutrophils and macrophages, and direct endothelial injury [15]. Angiotensin 2 is also responsible for regulating the collagen1 gene via mitogen-activated protein kinase/extracellular signal-regulated kinase 1/2 and transforming growth factor-β (TGF-β), the primary factors involved in fibrosis [15]. Consequently, uncontrolled production of metalloproteinases leads to epithelial and endothelial injury [16]. Lung fibrosis on computed tomography (CT) scans was depicted as ground-glass opacities, interstitial thickening, irregularity of the interface, and bands throughout the lung parenchyma [14].

About 25% of patients who survive ARDS will manifest evidence of restrictive lung disease on pulmonary function tests (PFTs) in the next six months from diagnosis [17].

New antifibrotic therapy can reduce the risk of pulmonary fibrosis in severe cases of COVID-19; however, there are ongoing clinical trials to determine the efficacy of novel antifibrotics [8]. When medical therapy no longer works, alternative treatments such as lung transplantation are considered to treat severe COVID-19 with ARDS [18].

This review provides an overview of pulmonary fibrosis resulting from COVID-19 infection, addressing the possible ongoing treatments to prevent early mortality and prolong the survival of these patients.

## Review

The presentations may vary from mild common cold to severe illness like SARS and middle east respiratory syndrome (MERS) [19,20]. ARDS is a major complication seen in critically infected patients with SARS-CoV-2 [21]. Few of the survivors of SARS-CoV-1 at follow-up presented with reduced exercise tolerance. They were observed to have fibrotic lung changes causing restrictive abnormalities [22]. Since there is a high resemblance between SARS-CoV-1 and SARS-CoV-2 infections, lung fibrosis may also be a long-term complication of COVID-19 pneumonia [23].

About 20% of the COVID-19 patients develop severe pneumonia, leading to SARS and multiple organ failure complications [24]. The fatality of the infection extensively increases with age, with 0.38% mortality below the age of 60 and 27% in those older [25]. Fatal cases may present as ARDS with significant alveolar damage causing loss and hyperplasia of type II pneumocytes cells, hyaline membrane formation, fibrin exudate along with alveolar thickening, and basement membrane damage [15]. Wu et al. described that 40% of patients who recovered from COVID-19 might develop ARDS consequently, and 20% of the ARDS patients may progress to pulmonary fibrosis [26].

Abdel-Hamid et al. conducted a prospective observational study on 85 moderately and severely affected COVID-19 patients and found that 38.5% of them have pulmonary residuals after three weeks. They concluded that male gender, high body mass index (BMI), high serum ferritin and C-reactive protein levels, low lymphocyte count, consolidation, and mixed consolidation/ground-glass opacities on initial CT scans are the independent predictors of post COVID- 19 pulmonary residuals [27]. 25% to 85% of patients can have remnant images compatible with pulmonary fibrosis on the chest images [17].

Advanced age, male gender, smoking, limited lung function, pre-existing comorbidities like diabetes, hypertension, cardiovascular disease, and obesity may impose a risk for developing severe COVID-19 [15,17].

The primary entry point of SARS-CoV-2 is via the epithelial cell of the nasal cavity, and from there, the infection further spreads to the respiratory system. The pulmonary epithelium has ACE-2 receptors on its surface, allowing the SARS-CoV-2 to enter the upper respiratory tract. Further, the descent of the virus to the lower respiratory tract infects the type-II alveolar cells of the lungs causing diffuse alveolar damage (DAD) [28,29]. Further progression of the disease results in collagen deposition at the site of lung injury resulting in extensive lung scarring and fibrosis [30,31]. Upregulation of MMP2, MMP8, and cathepsin proteins and downregulation of E-cadherin protein may also result in pulmonary fibrosis. Proteins like laminins, collagen VI, annexin A2, and fibronectin, which are the components of the extracellular matrix (ECM) of the basement membrane of the lung, are also downregulated [32].

TGF-β, a major pro-fibrotic stimulus, is directly amplified by the nucleocapsid protein of SARS- CoV-1. Since the nucleocapsid proteins of SARS-CoV-2 have a 90% similarity to SARS-CoV-1, it can be hypothesized as one of the possible mechanisms for lung fibrosis. TGF-β, along with connective tissue growth factor, is also upregulated by angiotensin II, which gets accumulated in the lungs due to the downregulation of ACE-2 caused by the virus [22].

Studies have also shown that IL-6 is involved in the fibrotic change of the lung. Severe COVID-19 patients receiving anti- IL-6 therapy may be at risk for developing pulmonary fibrosis [33]. Similarly, IL-1 also increased in COVID-19 patients has a fibrotic role [34].

Biomarkers related to pulmonary fibrosis

High-resolution CT (HRCT) and PFT are the most common methods of diagnosing and evaluating pulmonary fibrosis [34]. The serum biomarkers have been intensely researched to estimate additional modalities of predicting the severity, therapeutic responsiveness, and progression of any fibrotic process [34]. The pathological mechanisms of idiopathic pulmonary fibrosis (IPF) involve fibroblast proliferation and ECM remodeling, which determine a favorable environment for fibrotic scars formation. Selman et al. considered that not all inflammatory injuries could stimulate a fibrotic response of lung tissue [35]. Despite this statement, the recent research of Zhou et al. has suggested a significantly higher incidence of lung fibrosis in patients with severe or critical COVID-19 pneumonia than in patients with moderate COVID-19 [36].

The serum biomarkers of pulmonary fibrosis have been classified according to the mechanism driving the fibroproliferation: alveolocytes damage, including Krebs von den Lungen Antigen (KL-6), surfactant proteins A and D (SP-A, SP-D), chitinase-like protein (YKL-40); fibrogenesis, fibroproliferation, and matrix remodeling, including matrix metalloproteinases 1 and 7 (MMP1, MMP7), vascular endothelial growth factor (VEGF), insulin-like growth factor (IGF), lysyl oxidase-like 2 (LOXL-2), periostin, osteopontin, TGF-β; immune dysregulation, including CC motif chemokine ligand 18 (CCL 18), interleukin-6 (IL-6), and interleukin-8 (IL-8) [37,38]. However, despite many past or ongoing studies, specific criteria for evaluation of pulmonary fibrosis in post-COVID-19 patients have not been established yet [39].

Alveolar epithelial cells damage biomarkers

Krebs Von Den Lungen Antigen (KL-6)

KL-6 is a high-molecular-weight (200kDa) glycoprotein, categorized as a human transmembrane mucin 1 (MUC1), with a surface expression on type II pneumocytes provoked by the destruction of the air-blood barrier and its regeneration and therefore causing elevated serum concentration of this clinically important biomarker [40,41]. Thus, elevated serum KL-6 levels are associated with various respiratory diseases, particularly in ARDS, interstitial lung diseases (ILDs), or IPF [42]. The retrospective study of Peng et al. performed in 2020 profiled that higher serum concentrations of KL-6 have been observed in severe COVID-19 patients presenting signs of pulmonary fibrosis at discharge, which could be clinically significant in predicting fibrotic lung involvement [39,43].

Surfactant Proteins A and D (SP-A, SP-D)

Surfactant proteins A and D are sialoglycoprotein complexes synthesized and secreted by types II alveolar cells that reduce air-liquid interface tension and ensure local immunity. De Lara et al. obtained evidence that SP-A can downregulate DNA synthesis and inhibit the secretion of inflammatory mediators [44]. The cohort trial performed in Japan in 2020 has shown the efficacy of pirfenidone in IPF based on the reduction of serum concentration of SP-D, concluding that this biomarker might have a predictive and informative value [45]. 

Fibroproliferation and matrix remodeling biomarkers

Matrix Metalloproteinases 1 and 7 (MMP1 and MMP7)

MMPs are a family of zinc-dependent proteases responsible for degrading the ECM, playing a key role in pulmonary fibrosis. Proinflammatory cytokines could increase the expression of MMPs and, as a result, stimulate airway remodeling [46]. Tzoulevekis et al. have shown that MMP-7 concentration correlates with functional and clinical predictors of disease severity and mortality. It may be accurately used in distinguishing IPF from other chronic pulmonary diseases [47].

Immune dysregulation

Interleukin 6 (IL-6)

IL-6 is a pro-inflammatory and pro-fibrotic cytokine that induces the neutrophils’ activation and their accumulation at the injury site, consequently causing the release of proteases and oxygen-free radicals. Therefore, this pathway involves pulmonary interstitial edema and severe inflammatory response [36,48]. IL-6 is also considered a predictor of progression to severe COVID-19, which endorses the hypothesis that IL-6 receptor antagonists could control the cytokine storm induced by SARS-CoV-2 [36,48]. In a recent clinical trial (REMAP-CAP), it has been established that the anti-IL-6, tocilizumab, and sarilumab, could improve the evolution of critically ill patients with severe COVID-19 pneumonia [49]. The treatment with either tocilizumab or sarilumab and glucocorticosteroids in combination were more beneficial than the expected results for any intervention on its own, and the interaction between IL-6 receptor antagonists and glucocorticosteroids could be considered slightly synergistic but with substantial variability [49]. Although the results are encouraging regarding the 90-day survival, time to ICU, and hospital discharge, the tocilizumab group have brought attention to some adverse events, such as secondary bacterial infection, bleeding, cardiac events, and vision deterioration compared to arilumab with no serious adverse events reported [49].

The importance of the biomarkers mentioned above is indisputable in monitoring patients with post-COVID-19 pulmonary fibrosis, including their potential in early diagnosis and treatment responsiveness.

Novel antifibrotic drugs in patients with severe COVID-19 

Pulmonary fibrosis is one of the fatal complications in severe or critical COVID-19 patients [12,50]. Based on the resemblance of pulmonary fibrosis’ pathophysiological mechanisms between IPF and COVID-19 infection, it is considered that IPF regimens could be beneficial in COVID-19 pneumonia treatment. The clinical rationale of using antifibrotic therapy in COVID-19 patients is to prevent complications of ongoing infection, stimulate the recovering phase, and control the fibroproliferative processes [51]. Many early antifibrotic studies had concentrated on immunomodulatory system involvement, such as IFN-β and IFN-ɣ. However, the novel antifibrotic therapies should be focused on the fibrotic response following acute lung injury (ALI) rather than the new fibrotic lesions [8].

Some of the newly studied antifibrotic drugs target different molecules of the TGF-β pathway, including ɑvβ6 integrin (BG0011 [Biogen, Cambridge, MA]; PLN-74809 (Pliant Therapeutics, San Francisco, CA) and galectins (TD139 [Galecto Biotech, Copenhagen, Denmark]) [8]. Recent experimental data support the potential mechanism of these novel drugs in preventing the COVID-19 infection, based on the structure of SARS-CoV-2 spike proteins, particularly the Arg-Gly-Asp integrin-binding domain and the N-terminal galectin fold [8].

There is evidence that pirfenidone inhibits TGF-β-induced fibronectin synthesis and has antifibrotic and antiinflammatory properties, used to reduce the accumulation of inflammatory cells, fibroblast proliferation, and cytokine production and secretion [52]. Nintedanib is another antifibrotic drug approved by FDA for IPF treatment, known as a tyrosine kinase inhibitor, acting on fibroblast growth factor (FGF), platelet-derived growth factor (PDGF), and VEGF, and inhibiting the cascades of fibroblasts and myofibroblasts, additionally to a potential effect of pulmonary angiogenesis [53]. The INPULSIS trial has shown that Nintedanib reduces the decline of FVC in IPF, subsequently decreasing the disease progression with benefits seen by four to six weeks [17,54]. Nevertheless, Nintedanib should be used carefully due to an increased risk of bleeding and thrombosis caused by VEGF inhibition, consequently decreasing platelet activity and leukocyte adhesion. Furthermore, the PDGF blockage affects platelet production, possibly followed by thrombocytopenia [53]. SENCIS trial, a study to investigate the effects of Nintedanib on categorical changes in forced vital capacity (FVC) and other measures of ILD progression, has shown that subjects with systemic sclerosis-associated ILD (SSc‐ILD) have a clinically relevant benefit on the progression [55]. Both drugs, approved in by the FDA in 2014, have different mechanisms of action that attenuate the rate of lung function decrease (forced expiratory vital capacity or FEV1) and enhance life expectancy [12,16].

Recent studies have shown that mTOR’s protein-protein interaction could also be anti-COVID-19; consequently, the anti-mTOR rapamycin’s use might be adjusted [8]. A double-blind phase 2 clinical trial completed in April 2021 investigated the role of ACE2 in COVID-19 infection, specifically the impact of ADAM17 in the hydrolysis of AngII to Ang1-7 [8,56].

A phase 1 clinical trial was conducted in 2020 in Wuhan City, China on 27 COVID‐19 patients that received intravenous transfusion (IV) of human embryonic stem cells-derived immunity‐ and matrix‐regulatory cells (hESC‐IMRCs) [50]. It demonstrated safe IV use of hESC‐IMRCs for pulmonary fibrosis in COVID‐19 patients with clinical improvement within a short period after IV hESC‐IMRC transfusions. Additionally, they observed safety in long‐term follow‐up at a later stage in 100% of cases (27/27 patients) [50].

Clinical trials are ongoing to find adequate therapy for pulmonary sequelae such as post-COVID-19 fibrosis of the lungs. Four of them are in phase 4 (Table 1) [57-60].

**Table 1:** Ongoing clinical trials of anti-fibrotic therapy and COVID-19 (Phase 4)

Clinical Trial number	Location	Status	Intervention
NCT04818489 [57]	Egypt	Phase 4, recruiting	Drug: Colchicine 0.5 MG
NCT04912011 [58]	Poland	Phase 4, recruiting	Drug: Canrenoate Potassium
NCT04619680 [59]	USA	Phase:4, recruiting	Drug: Nintedanib
NCT04856111 [60]	India	Phase 4	Drug: Pirfenidone Drug: Nintedanib

Despite the benefits of the novel antifibrotic therapies in severe COVID-19, further trials are required to investigate these regimens' long-term efficacy and safety.

Alternative treatment

The treatment for COVID-19 patients with severe pulmonary involvement should be a multidisciplinary decision [18]. Patients with end-stage lung disease have lung transplantation as a life-preserving treatment. It is not often indicated in ARDS related to infectious causes. However, Bharat et al. have reported a multicenter study of successful lung transplant procedures in 11 out of 12 critically ill COVID-19 patients who had not recovered even after proper medical management and were at high risk of dying [18]. They showed ongoing ALI with aspects of lung fibrosis in pathological findings. The majority were male (9/12) with a median age of 48 years old, and BMI median was 29.5 kg/m^2^ (IQR 24·8-26·8). On the 30th-day post-surgery, 100% of the patients were alive, compared to lung transplant patients with non-COVID-19-related terminal illness lung diseases (USA 30-day survival 97·7%). Eleven out of 12 remained alive and recovering well after a median follow-up of 80 days (32-160). They suggested a transplantation decision for patients with lung injury due to severe COVID-19 disease who would probably not survive, younger than 65 years old with no pre-existing comorbidities or manageable comorbidities [18].

There is much to be explored, and future studies about the effects of lung transplants in patients with severe SARS-CoV-2 disease with no response to other treatments should be performed.

Rehabilitation

About 14% of COVID-19 patients experience severe disease, and 6% develop critical illness. According to the most recent clinical guidelines, pulmonary rehabilitation could improve physical and psychological conditions, including exercise training, education, and behavioral changes [61]. Patients who underwent respiratory illness and mixed respiratory and surgical populations could improve muscle strength, walking, and functional ability with significant positive effects in the six minutes walking test (6MWT) and Barthel index [62]. Pulmonary rehabilitation has been applied with positive results in severe cases with pulmonary fibrosis [61].

## Conclusions

Lung fibrosis is one of the major long-term complications in patients with COVID-19. Furthermore, risk factors like advanced age with limited lung function, preexisting comorbidities, such as diabetes, cardiovascular disease, hypertension, and obesity increase the risk of developing fibrotic lung changes in survivors who presented with reduced exercise tolerance.

Biomarkers related to pulmonary fibrosis such as KL-6, SP-D, MMP-7, IL-6 have a great predictive potential in early diagnosis and treatment responsiveness in patients with post-COVID-19 pulmonary fibrosis. Anti-fibrotic drugs, such as Nintedanib and Pirfenidone, are under clinical trials. A detailed follow-up and protocols for rehabilitation should be encouraged to improve the quality of life in such patients.
